# Systematic identification of tRNA genes in *Drosophila melanogaster*

**DOI:** 10.17912/micropub.biology.000560

**Published:** 2022-05-01

**Authors:** Steven J Marygold, Patricia P Chan, Todd M Lowe

**Affiliations:** 1 FlyBase, Department of Physiology, Development and Neuroscience, University of Cambridge, Cambridge, CB2 3DY, UK; 2 Department of Biomolecular Engineering, University of California Santa Cruz, CA 95064, USA

## Abstract

Transfer RNAs (tRNAs) are ubiquitous adapter molecules that link specific codons in messenger RNA (mRNA) with their corresponding amino acids during protein synthesis. The tRNA genes of Drosophila have been investigated for over half a century but have lacked systematic identification and nomenclature. Here, we review and integrate data within FlyBase and the Genomic tRNA Database (GtRNAdb) to identify the full complement of tRNA genes in the *D. melanogaster* nuclear and mitochondrial genomes. We apply a logical and informative nomenclature to all tRNA genes, and provide an overview of their characteristics and genomic features.

**Figure 1. Drosophila tRNA genes:**
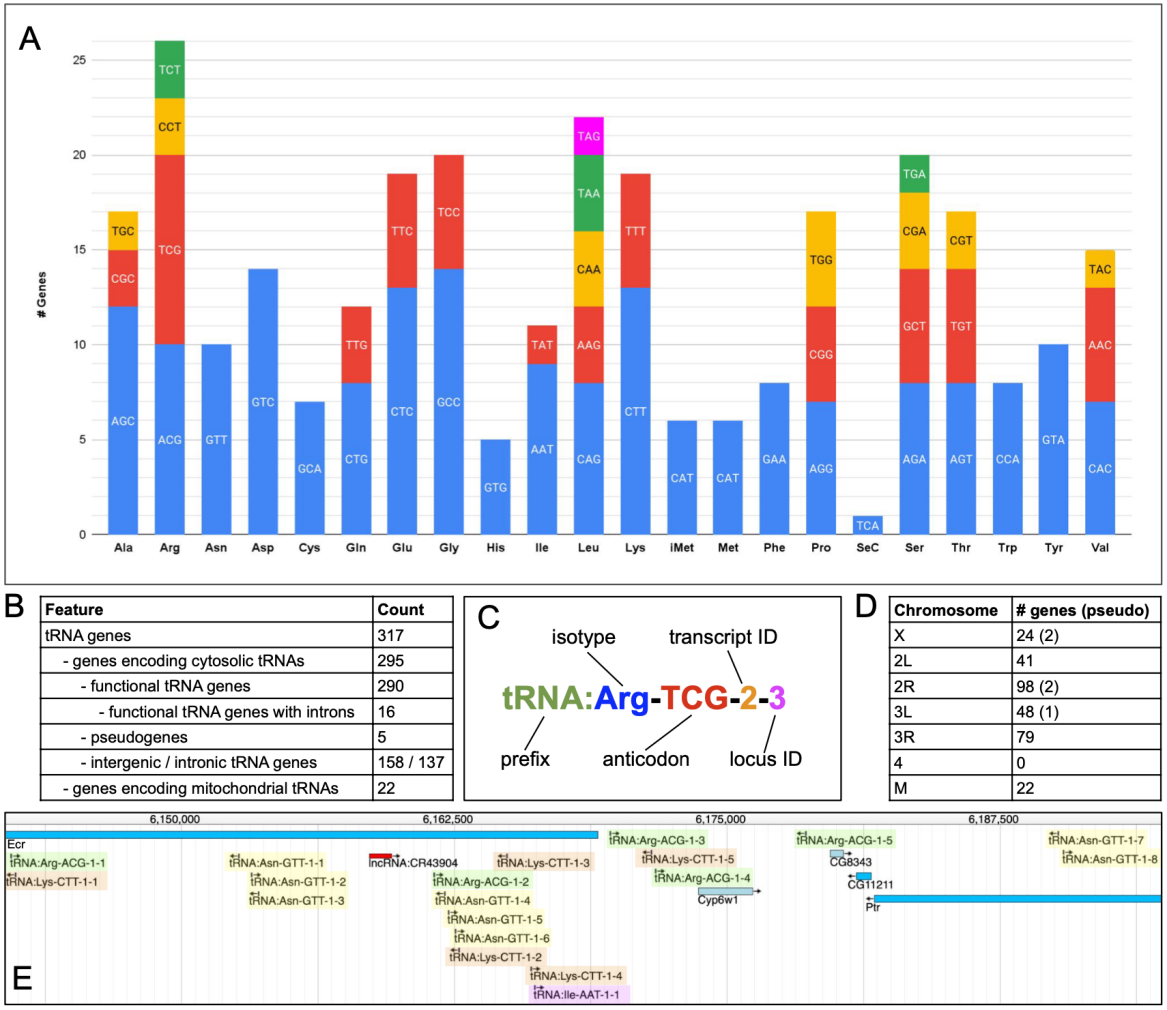
(A) Isoacceptor counts of functional cytosolic tRNA genes in Drosophila - pseudogenes and mitochondrial tRNA genes are excluded. Colors distinguish distinct anticodons within each isoacceptor family. Counts for initiator (iMet) and elongator tRNA:Met are shown separately. (B) Summary statistics for all Drosophila tRNA genes. (C) Example of tRNA gene nomenclature syntax. (D) Distribution of tRNA genes across major chromosomal scaffolds; pseudogene numbers are in parentheses. ‘M’ = mitochondrial genome. (E) Example of a tRNA gene cluster from the 2R:6,144,000..6,195,000 genomic region, corresponding to cytological region 42A shown in Figure 3 of Kubli 1982. (Notably, the only significant change to tRNA annotations in this region is the addition of tRNA:Arg-ACG-1-4 in the current version.) Green highlight: tRNA:Arg-ACG-1 isodecoders; orange highlight: tRNA:Lys-CTT-1; yellow highlight: tRNA:Asn-GTT-1; magenta highlight: tRNA:Ile-AAT-1; blue rectangles: protein-coding genes; red rectangle is a lncRNA gene.

## Description

tRNAs are universal to all cellular life and provide the essential molecular link between mRNA codons and their corresponding amino acids during translation (reviewed by Suzuki 2021). The main functional regions in a tRNA are the anticodon triplet, which base pairs with mRNA codons, and the 3′ end to which the cognate amino acid is attached. Codon degeneracy for the 21 amino acids (20 standard amino acids plus selenocysteine) means that up to six tRNAs with distinct anticodons (‘isoacceptors’) are required depending on the amino acid. tRNA diversity is further increased through the existence of tRNAs that share the same anticodon but differ in the sequence of their body structure (Goodenbour and Pan 2006). Such ‘isodecoders’ may differ from each other by just one or several nucleotides. Moreover, each specific isodecoder sequence can be present in multiple copies within a genome. This combination of diversity and redundancy results in eukaryotic nuclear genomes having hundreds of genes encoding tRNAs functioning in cytosolic translation (cytosolic tRNAs). An additional set of tRNAs functioning in mitochondria are encoded by the mitochondrial genome of eukaryotes: in vertebrates and many other metazoa, there are often 22 mitochondrial tRNA genes with tRNA:Leu and tRNA:Ser represented by two different isoacceptors.

The tRNAs and tRNA genes of *Drosophila melanogaster* (hereafter ‘Drosophila’) have been investigated for over half a century. Early work identified the sequences and locations of many individual cytosolic tRNA genes, demonstrating that several isoacceptor families appear as clusters in cytological (polytene chromosome) views of the genome (reviewed in Kubli 1982 and Sharp *et al.* 1983). Subsets of mitochondrial tRNA genes were also reported (de Bruijn 1983; Garesse 1988). Subsequently, the analysis of the sequenced Drosophila genome predicted 292 cytosolic tRNA genes (Adams *et al.* 2000). This number was later refined in release 4 of the genome to 297 cytosolic tRNA genes, including four pseudogenes (Drosophila 12 Genomes Consortium 2007; Bergman and Ardell 2014). However, none of these studies fully classified or specifically named tRNA genes by their molecular features, and the genomic data were not fully rationalized with the earlier information on tRNAs present within FlyBase, the primary database for Drosophila research (Larkin *et al.* 2021).

We revisited cytosolic tRNA gene annotations in the current version of the Drosophila genome (release 6), integrating gene predictions from the Genomic tRNA database (GtRNAdb; Chan and Lowe 2016; Chan *et al.* 2021) with existing data within FlyBase. We find there are a total of 295 cytosolic tRNA genes, of which 289 encode tRNAs decoding the 20 standard amino acids, one encodes a selenocysteine tRNA, and five are classified as tRNA-like genes/pseudogenes (Figure 1A, 1B; Extended Data Table 1). Importantly, this rationalization exercise resulted in improvements within both databases. For example, ~20 records pertaining to unlocalized tRNA genes were merged/deleted within FlyBase, two tRNAs with undetermined isotypes within the GtRNAdb were corrected, and pseudogene classifications within both databases were made consistent.

Prior to our analysis, greater than 50% of Drosophila cytosolic tRNA genes were unnamed in FlyBase, while the named genes used an ambiguous and esoteric nomenclature incorporating the single letter amino acid code and cytogenetic map information but lacking anticodon information. We therefore implemented the logical and systematic nomenclature used by the GtRNAdb within FlyBase (Figure 1C; Extended Data Table 1). This syntax comprises the 3-letter code of the cognate amino acid (isotype), the anticodon triplet, a number identifying each unique transcript (isodecoder) sequence, followed by a second number to specify each copy (locus) of that sequence within the genome. This is preceded by the standard ‘ *tRNA:* ’ prefix used for tRNA genes in FlyBase. For example, *tRNA:Arg-TCG-2-3* is the systematic name given to the gene encoding the third copy of the second unique transcript of tRNA:Arg-TCG. tRNA pseudogenes are named using the standard FlyBase syntax for pseudogenes, with a greek Psi character appended to the gene name, e.g. *tRNA:His-GTG-2-1Ψ* .

Mitochondrial tRNA genes are not currently included within the GtRNAdb. We therefore compared existing FlyBase annotations against Drosophila entries in the mitotRNAdb (Jühling *et al* . 2009) and verified these using the tRNAscan-SE program (Chan *et al* . 2021). This resulted in minor edits to five mitochondrial tRNA sequences in FlyBase. We also applied the standardized nomenclature to the 22 mitochondrial tRNA genes, including the standard ‘ *mt:* ’ prefix used for genes of the mitochondrial genome in FlyBase (Figure 1B; Extended Data Table 2).

Extended Data Tables 1 and 2 provide detailed information on all the cytosolic and mitochondrial tRNA genes, respectively. Among the functional, cytosolic tRNAs decoding the 20 standard amino acids, each isoacceptor is encoded by between five (tRNA:His) and 26 (tRNA:Arg) genes, with the number of distinct anticodons in each isoacceptor family ranging from one (e.g. tRNA:Asn-GTT) to five (tRNA:Leu-CAG, tRNA:Leu-AAG, tRNA:Leu-CAA, tRNA:Leu-TAA, tRNA:Leu-TAG). Up to four distinct transcript sequences exist for a given anticodon (as is the case for tRNA:Arg-TCG, tRNA:Cys-GCA, tRNA:Gln-CTG and tRNA:Leu-TAA), and a given transcript sequence may be present in up to 13 exact gene copies (tRNA:Gly-GCC-1 and tRNA:Lys-CTT-1). Overall, there are 44 different isoacceptors and 84 distinct tRNA transcripts encoded by the Drosophila nuclear genome. Cytosolic tRNA genes are present on all major chromosome arms (Figure 1D) and are frequently found within clusters, harboring members of either the same or different isoacceptor families (Figure 1E; Kubli 1982; Phillips and Ardell 2021). 46% of cytosolic tRNA genes are located within introns of protein-coding genes, with the remainder being intergenic. A minority (5%) of cytosolic tRNA genes contain an intron, namely two tRNA:Ile-TAT, four tRNA:Leu-CAA and ten tRNA:Tyr-GTA genes (Figure 1B; Bergman and Ardell 2014). These characteristics are largely comparable with the cytosolic tRNA gene complement of other metazoa ( http://gtrnadb.ucsc.edu/ ). However, the Drosophila genome is distinguished by its relative paucity of non-functional tRNA-like genes: <2% of Drosophila tRNA genes are classed as pseudogenes or repetitive element derivatives, compared to 20% in *C. elegans* , 30% in humans or >99% in rodents.

Notably, this project enabled several additional improvements to the representation of tRNAs within FlyBase. All functional (Gene Ontology) annotations were reviewed and revised as necessary. Reciprocal links between tRNA gene/transcript reports in FlyBase and corresponding pages at the GtRNAdb and RNAcentral (RNAcentral Consortium 2021) have been established, and 2D structural images for Drosophila tRNAs (Sweeney *et al.* 2021) have been added. Finally, a ‘Gene Group’ report (Attrill *et al.* 2016) for the Drosophila tRNA genes has been generated ( https://flybase.org/reports/FBgg0000459.html ), which provides easy access to all classes of tRNA gene and their associated data within FlyBase.

In conclusion, we have generated definitive sets of cytosolic and mitochondrial tRNA genes present in the Drosophila genome and implemented a systematic and informative nomenclature for them. The improved datasets are available from several databases, including FlyBase, GtRNAdb, RNAcentral, and the Alliance of Genome Resources (Alliance of Genome Resources Consortium 2022). Our work will facilitate further exploration of tRNA biology within Drosophila as well as new comparative studies with other species.

## Methods

Data on cytosolic tRNA genes were accessed and downloaded from FlyBase ( http://flybase.org ) and the GtRNAdb ( http://gtrnadb.ucsc.edu/ ). Data were initially compared and rationalized between FlyBase release FB2015_04 and GtRNAdb release 16. Necessary revisions were made in subsequent database releases, and the data presented herein are from FlyBase release FB2022_02 and GtRNAdb release 19 (which uses tRNAscan-SE 2.0). tRNAscan-SE uses multiple score thresholds to discriminate functional tRNA genes from likely non-functional tRNA-like genes/pseudogenes based on their primary sequence, secondary structure and isotype-specific models (Chan *et al.* 2021).

Data on mitochondrial tRNA genes were accessed and downloaded from FlyBase ( http://flybase.org ) and mitotRNAdb http://mttrna.bioinf.uni-leipzig.de/mtDataOutput/ . Additionally, the Drosophila mitochondrial genome (RefSeq NC_024511) was used as a query sequence in tRNAscan-SE 2.0 ( http://trna.ucsc.edu/tRNAscan-SE/ ) with parameters ‘other mitochondrial’ as the sequence source, ‘Invertebrate Mito’ as the genetic code and a score cutoff of zero. Necessary revisions were made in FlyBase and data presented herein are from FlyBase release FB2022_02.

## Extended Data

Description: Cytosolic tRNA genes. Resource Type: Dataset. DOI: 10.22002/D1.20161

Description: Mitochondrial tRNA genes. Resource Type: Dataset. DOI: 10.22002/D1.20162
